# Live-cell fluorescence imaging of ciliary dynamics

**DOI:** 10.52601/bpr.2021.210005

**Published:** 2021-04-30

**Authors:** Quanlong Lü, Chuanmao Zhang, Christopher J. Westlake

**Affiliations:** 1 Laboratory of Cellular and Developmental Signaling, Center for Cancer Research, National Cancer Institute, National Institutes of Health, Frederick, MD 21702, USA; 2 Ministry of Education Key Laboratory of Cell Proliferation and Differentiation, College of Life Sciences, Peking University, Beijing 100871, China

**Keywords:** Cilia, Ciliogenesis, Ciliary dynamics, Live-cell fluorescence imaging

## Abstract

The cilium was one of the first organelles observed through a microscope. Motile cilia appear as oscillating cell appendages and have long been recognized to function in cell motility. In contrast, the far more widespread non-motile cilia, termed primary cilia, were thought to be vestigial and largely ignored following their initial description over a century ago. Only in the last two decades has the critical function of primary cilia been elucidated. Primary cilia play essential roles in signal transduction, chemical sensation, mechanosensation and light detection. Various microscopy approaches have been important for characterizing the structure, dynamics and function of the cilia. In this review, we discuss the application of live-cell imaging technologies and their contribution to our current understanding of ciliary processes.

## INTRODUCTION

In eukaryotic cells, there are two types of cilia, motile cilia and primary cilia. Cilia are small organelles, several microns long and less than half a micron wide (Flaherty *et al*. 2020). Transmission electron microscopy studies revealed that the main structural difference between these two types of cilia is the presence of a central pair of microtubules in motile cilia, which is important for movement. Historically, motile cilia have been extensively studied in lung epithelial cells, brain ependymal cells, and sperm tails of mammals. On the other hand, non-motile primary cilia were thought to be vestigial organelles in higher-order animals (Satir and Christensen 2008). However, over the last two decades, primary cilia have attracted considerable attention as defects in their function have been linked to multiple human genetic diseases and developmental disorders termed ciliopathies, including Bardet-Biedl syndrome, Joubert syndrome, Leber congenital amaurosis, Mckusick-Kaufman syndrome, and polycystic kidney disease.

Motile and primary cilia share common basic structures, including the centriole/basal body, the microtubule-based core axoneme, the transition zone, and the ciliary membrane surrounding the axoneme. The basal body is composed of nine microtubule triplets that convert to nine microtubule doublets in the axoneme. The transition zone is a structural intermediate between the basal body and the axoneme controlling traffic in/out of the cilium. The ciliary membrane is docked on the transition fiber of the basal body and is continuous with the plasma membrane, although it has a distinct lipid and protein composition partly established by the transition zone (Emmer *et al*. 2010; Musgrave *et al*. 1986; Tyler *et al*. 2009).

Advances in microscopy have led to a new understanding of the cilium structure and function. Cilia were initially described as "little legs, which moved very nimbly" by Leeuwenhoek in 1675 as he observed protozoa using a light microscope (Satir 1995). Zimmermann subsequently discovered primary cilium in mammalian cells, which he called central flagella in 1898 (Zimmermann 1898). The development of transmission electron microscopy (TEM) provided the first insights into the cilium structure. Using TEM in rat lungs, Sorokin first characterized the cilia assembly process known as ciliogenesis and described this structure as the primary cilium (Sorokin 1968). Although TEM was important for clarifying the cilium ultrastructure, primary cilia function remained elusive. Over the past two decades, genetic studies and analysis with electron microscopy (EM) and light microscopy have shed light on both cilium structure and function. Here, we discuss the application of different live-cell imaging modalities in investigating ciliary processes and technical considerations when applying this approach to study cell biological phenomenon.

## HISTORICAL PERSPECTIVE ON LIVE-CELL IMAGING OF CILIA

Live-cell microscopy is an indispensable technique for studying cilium function and dynamics. Intraflagellar transport (IFT) along the axoneme was first discovered using live-cell imaging. Early live-cell imaging using video-enhanced differential interference contrast (DIC) microscopy revealed the rapid bidirectional movement of granule-like particles along the length of flagella termed IFT in *Chlamydomonas* (Kozminski *et al*. 1993). This approach effectively visualizes IFT in the *Chlamydomonas* flagella extending well beyond the cell body without interference from other cellular structures, but smaller primary cilia are typically obscured (Ishikawa and Marshall 2015). More recently, fluorescently tagged proteins have been used for live-cell imaging following the discovery of green fluorescent protein (GFP) and other spectral fluorescence proteins. Additionally, self-labeling proteins that are covalently labeled with fluorescent chemical ligands offer excellent spectral, spatial, and temporal flexibility for live-cell imaging (Crivat and Taraska 2012). These labeling techniques, combined with live-cell imaging, have enabled the monitoring of real-time IFT particle movement not only in single-celled *Chlamydomonas* but also highly specialized mammalian cells and helped establish a critical role for IFT in establishing primary cilium structure and function (Bloodgood 2009; Diener 2009; Follit *et al*. 2006; Ishikawa and Marshall 2015).

**Figure 1:**
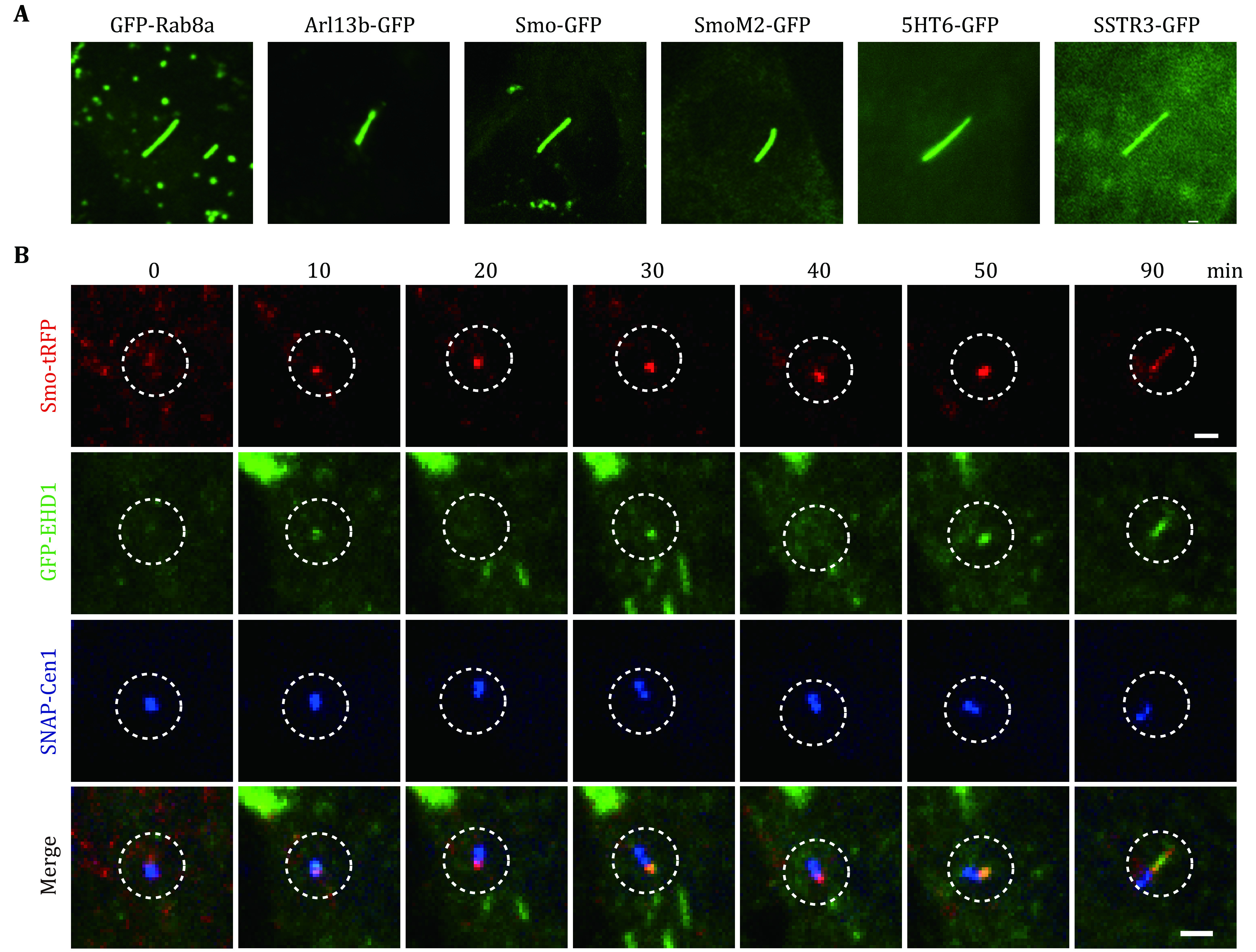
Ciliary markers and ciliogenesis in RPE-1 cells. **A** GFP-tagged Rab8a, Arl13b, SMO, SmoM2, 5HT6 and SSTR3 localize to primary cilia in RPE-1 cells. Images are captured in live cells with epifluorescence microscopy. Scale bar, 2 µm. **B** Ciliary membrane assembly was imaged using spinning disk confocal microscopy. SMO-tRFP and SNAP-Centrin1 mark the ciliary membrane and centrosome, respectively. SNAP-centrin1 was stained with SNAP-Cell 647-SiR for 30 min before imaging. Live-cell imaging demonstrates the dynamics of GFP-EHD1 localization in the developing ciliary membrane. Image series show the ciliary membrane assembly process from vesicle docking to cilia elongation. Images were captured every 10 min. Scale bar, 2 µm

Live-cell imaging has also proven to be a powerful tool for studying cilium assembly and disassembly. Key to these investigations was the identification of fluorescently-tagged proteins associated with ciliogenesis. The axoneme protein tubulin and associated or embedded ciliary membrane proteins have been used to mark cilia for live imaging. Fluorescently-labeled tubulin was used for live imaging of the apical membrane-localized primary cilium in polarized cells (Ott and Lippincott-Schwartz 2012). In non-polarized cells, where the cilium can be found at the bottom of or lateral to the cell body, fluorescent tubulin signals may be obscured by its fluorescence signals in the cell body. As an alternative, multispanning transmembrane domain-containing proteins with ciliary targeting motifs fused to fluorescent tags allow ciliogenesis monitoring in live cells (Ott and Lippincott-Schwartz 2012). These proteins include somatostatin receptor 3 (SSTR3), 5-hydroxytryptamine receptor 6 (5HT6), melanin-concentrating hormone receptor 1 (MCHR1), and smoothened (Smo) (Fu *et al*. 2016; Hu *et al*. 2017; Iwanaga *et al*. 2011; Phua *et al*. 2017). Membrane trafficking regulators important for cilium assembly, including the small GTPase Arl13b and Rab8, can also be used to visualize the cilium in live-cell imaging (Mazo *et al*. 2016; Westlake *et al*. 2011) (Fig. 1).

The cilium is dynamically regulated during cell cycle progression. It assembles in G1 or G0 and disassembles prior to mitosis (Plotnikova *et al*. 2009; Zhou 2009). The timing of cilia assembly and disassembly is critical for cell proliferation and differentiation during development. In cultured mouse kidney cells, primary cilium dynamics during the cell cycle were tracked live with confocal microscopy (Piotrowska-Nitsche and Caspary 2012). Using SSTR3-GFP to mark cilia in murine inner medullary collecting duct (mIMCD3) cells, cilium formation occurs asynchronously between the resulting daughter cells following mitosis. In other cultured cells, ciliogenesis can also be induced by removal of serum and observed by live-cell imaging (Lu *et al*. 2015b; Santos and Reiter 2008; Westlake *et al*. 2011).

**Figure 2:**
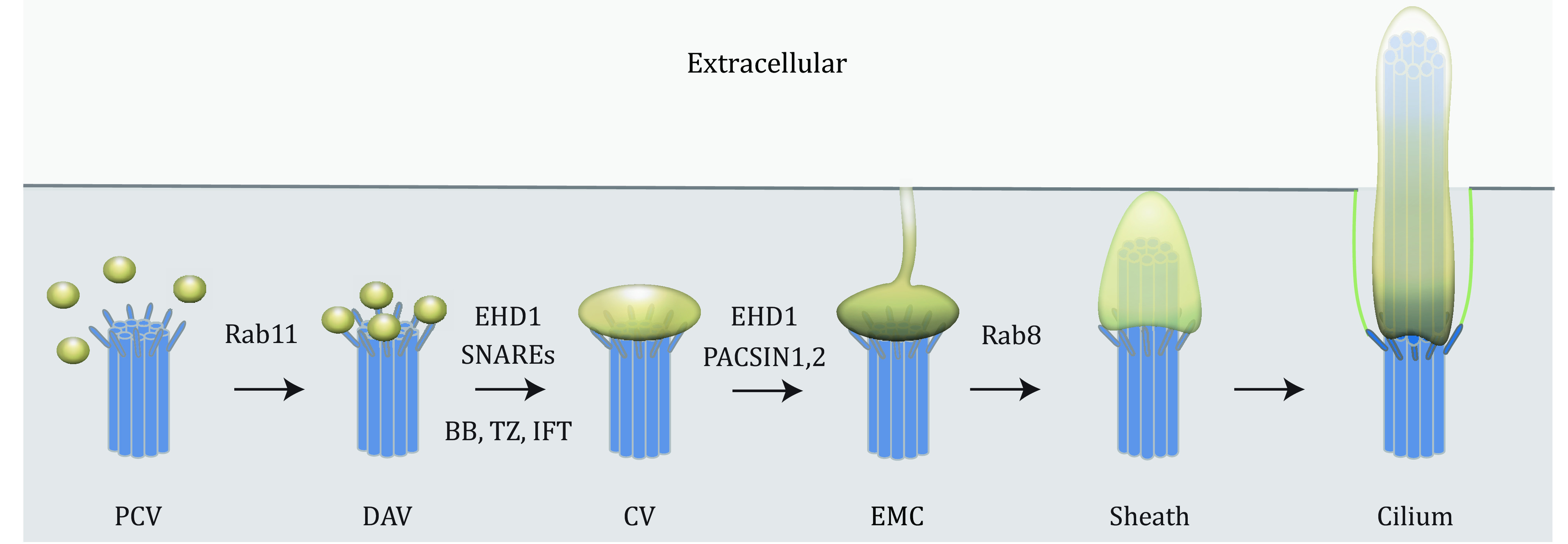
Sequential steps of primary cilium assembly revealed by live-cell imaging. Model of intracellular ciliogenesis. At the early stage of ciliogenesis, pre-ciliary vesicles (PCVs) are transported to the mother centriole through a Rab11-mediated membrane trafficking pathway. Some of these vesicles dock onto the mother centriole's distal appendages and become distal appendage vesicles (DAVs). EDH1 and SNARE proteins promote the reshaping and fusion of DAVs to form a larger ciliary vesicle (CV). The reorganization of membrane vesicles from DAV to CV is a prerequisite step for the basal body (BB) and transition zone (TZ) formation and IFT proteins recruitment. Remarkably, EHD1 and PACSIN1/2 assemble membrane tubules from the developing intracellular cilium that can connect to the plasma membrane and create an extracellular membrane channel (EMC) to the outside of the cell. Rab8 promotes ciliary sheath growth from the CV and the intracellular ciliary sheath subsequently fuses with the plasma membrane and the exposing the developing cilium is exposed to the extracellular environment. The sequential assembly of CV, BB, TZ and IFT was revealed by live-cell imaging (Lu *et al*. 2015a). The application of live-cell imaging shed new light on mechanisms of ciliogenesis regulation

Ciliogenesis is a complex multi-step process requiring the docking of the centriole to cellular membranes, initiating a centriole to basal body transition, followed by transition zone formation and IFT recruitment needed to assemble the axoneme. In some cells, the centrioles dock directly to the plasma membrane (extracellular pathway) while others partially assemble the cilium inside the cell (intracellular pathway). In the intracellular pathway, small membrane vesicles (~50 nm) dock at the mother centriole and assembly into a larger (300–500 nm) ciliary vesicle (CV), which then organizes into a double membrane sheath as the axoneme is constructed and ultimately fuses with the plasma membrane. Each of these stages has been analyzed with live-cell imaging in cultured cells expressing various markers of ciliary assembly (Lu *et al*. 2015b). Centriolar markers (*i.e*., GFP-CP110), transition zone proteins (*i.e*., B9D2-GFP), IFT protein (*i.e*., IFT20-GFP) can be co-expressed with ciliary membrane markers such as Rab8a, EHD1, Smo and 5HT6-tRFP to examine the order of ciliogenesis events based on targeting to the developing cilium. For example, live-cell imaging analysis confirmed that the membrane reorganization by EHD1 is a prerequisite step for basal body establishment, transition zone formation and IFT20 recruitment. Furthermore, Rab8 recruitment to the developing cilium membrane is observed after these steps. These observations suggest chat proper membrane association serves as a checkpoint to ensure early ciliogenesis progression. Hence, only following IFT20 and TZ proteins recruitment does coordinated axoneme and ciliary membrane growth occur. Live-cell imaging along with genetic knockdown and TEM studies revealed that CV formation is critical to establish the basal body and transition zone and to recruit IFT at the early stages of ciliogenesis (Fig. 2) (Lu *et al*. 2015b).

## CONSIDERATIONS FOR LIVE-CELL IMAGING OF CILIA

Live-cell fluorescence imaging approaches can be used to reduce artifacts associated with fixation and antibody-based immunostaining. However, artifacts associated with overexpression, the effect of fluorescence tag on protein behavior, and cell autofluorescence must be considered in these studies. Care must also be taken to ensure cells are healthy during imaging. In particular, phototoxicity arising from light excitation of fluorescent molecules can affect the health of cells in live imaging studies. These free radicals are highly reactive and can be destructive to cellular components (Frigault *et al*. 2009). The effects of phototoxicity can be limited by choosing bright and stable fluorescent tags, which can help to reduce the exposure of cells to damaging light while ensuring high signal-to-noise ratios when capturing images. The brightness of the fluorophore will also affect the length of time, samples can be imaged before photobleaching of molecules occurs.

Other considerations for choosing fluorophores for live imaging include their solubility, toxicity, stability, dimerization/aggregation properties and maturation time, and cell permeability for dyes. The distribution of the fusion protein should also be compared to the endogenous protein to ensure proper localization. Moreover, efforts should be made to express fluorescent-labeled proteins at levels comparable to native protein levels to avoid issues with exogenous proteins affecting other cellular functions. This can be accomplished by maintaining exogenous protein levels similar to endogenous expression through the use of an appropriate promoter, employing an inducible expression system, or stably integrating a single gene copy using the FLP-IN system (Jensen *et al*. 2020; Qin *et al*. 2010).

Fluorescent protein tags like GFP, Emerald, YFP, mCherry, mCerulean3, TagRFP, and TdTomato have been successfully applied in live-cell imaging of cilia (Phua *et al*. 2017; Toro-Tapia and Das 2020; Trivedi *et al*. 2012; Ye *et al*. 2013; Zhang *et al*. 2019). Non-fluorescent self-labeling proteins like HaloTag, SNAP-tag and CLIP-tag can also be applied in live-cell imaging (Gautier *et al*. 2008; Los *et al*. 2008). These tags can covalently bind to synthetic ligands, which can be modified with a variety of fluorophores for imaging. Different fluorescent proteins can be used together to mark different genes for multi-color live-cell imaging. For example, we have successfully expressed GFP-EHD1, Smo-tRFP, and SNAP-centrin1 simultaneously in human retinal pigment epithelial (RPE-1) cells and identified a novel tubular membrane structure that connects to the developing primary cilium (Fig. 1B and Fig. 2) (Insinna *et al*. 2019b). In some cases, it is necessary to have higher brightness or reduced photobleaching of the cilia marker for live-cell imaging studies. In such instances, multiple copies of fluorescent protein molecules can be concatenated to enhance the signal of specific target proteins (Tanenbaum *et al*. 2014). It has been reported that some ciliary proteins can be tagged with up to 14 copies of GFP without affecting the ciliary structure and function in *C. elegans* (Xie *et al*. 2020). However, as mentioned, with high fluorescent molecule expression comes concerns about artifacts for specific proteins (Guadiana *et al*. 2013).

It is also important to select suitable cell lines for live-cell imaging of cilia. Because light microscopy has worse axial resolution than lateral resolution, it is better to select cilia growing laterally to image (Fouquet *et al*. 2015). Polarized epithelial cells like murine inner medullary collecting duct (mIMCD3) cells or Madin-Darby canine kidney (MDCK) cells usually have cilia growing upright at the apical surface (Rangel *et al*. 2019). Some long cilia may be positioned right along the coverslip and these should be selected when imaging ciliary dynamics. Human retinal pigment epithelial (RPE-1) cells, human fibroblasts (HF), mouse embryonic fibroblasts (MEFs) and 3T3 cells, have most of their cilia growing on the dorsal or ventral side of the cell, which is useful for many applications when study cilia dynamics with live-cell imaging (Ocbina and Anderson 2008; Ott and Lippincott-Schwartz 2012; Shimada *et al*. 2017). RPE-1, HF, 3T3 and MEF cells form short cilia inside the cell before docking to the plasma membrane. These cells can be used to study intracellular ciliogenesis (Lu *et al*. 2015b; Shimada *et al*. 2017). In cultured RPE-1 cells, a large portion of cilia are growing at the bottom of the cell. These cilia are ideal for TIRF microscopy imaging (Shimada *et al*. 2017).

## LIVE-CELL IMAGING MODALITIES FOR CILIA

Here, we will discuss live-cell imaging modalities used to investigate ciliogenesis processes, epifluorescence microscopy, laser scanning confocal microscopy (LSCM), spinning disc confocal microscopy (SDCM), total internal reflection fluorescence microscopy (TIRFM), and fluorescence recovery after photobleaching (FRAP) microscopy. A comparison of the features and applications of these microscopies in cilia research is listed in Table 1. The pros and cons of these live-cell imaging approaches are also discussed below.

**Table 1:** A comparison of the features and applications of the microscopies in cilia research

Microscopes	Features	Applications in the cilia field	Specimens
Widefield	The entire specimen field is illuminated by a light source, high out-of-focus background, Images are captured by cameras	Cilium assembly and disassembly; Ciliary trafficking	Thin cells
LSCM	The specimen is illuminated by a single laser beam in a point-by-point scanning pattern, high excitation intensity, low out-of-focus background at the expense of speed; Signal intensity is detected by photomultiplier	Cilium assembly and disassembly; Ciliary trafficking; Ciliary ectosomes	Thin cells or thick tissues
SDCM	The entire specimen field is illuminated by multiple excitation beams simultaneously, low excitation intensity, rapid spatial and temporal image acquisition, low out-of-focus background; Images are captured by cameras	Cilium assembly and disassembly; Ciliary trafficking; Ciliary ectosomes; IFT movement	Thin cells or thick tissues
TIRFM	Only a thin surface region of a specimen is illuminated, lowest out-of-focus background, Excellent axial resolution; Images are captured by cameras	Ciliary trafficking; IFT movement	Thin cells
FRAP	Only selected ROI is intensively illuminated to photobleach fluorophores, the diffusion of fluorophores to the bleached region can be imaged with confocal or widefield setups	Ciliary trafficking; Intraciliary transport	Thin cells

### Epifluorescence microscopy

**Figure 3:**
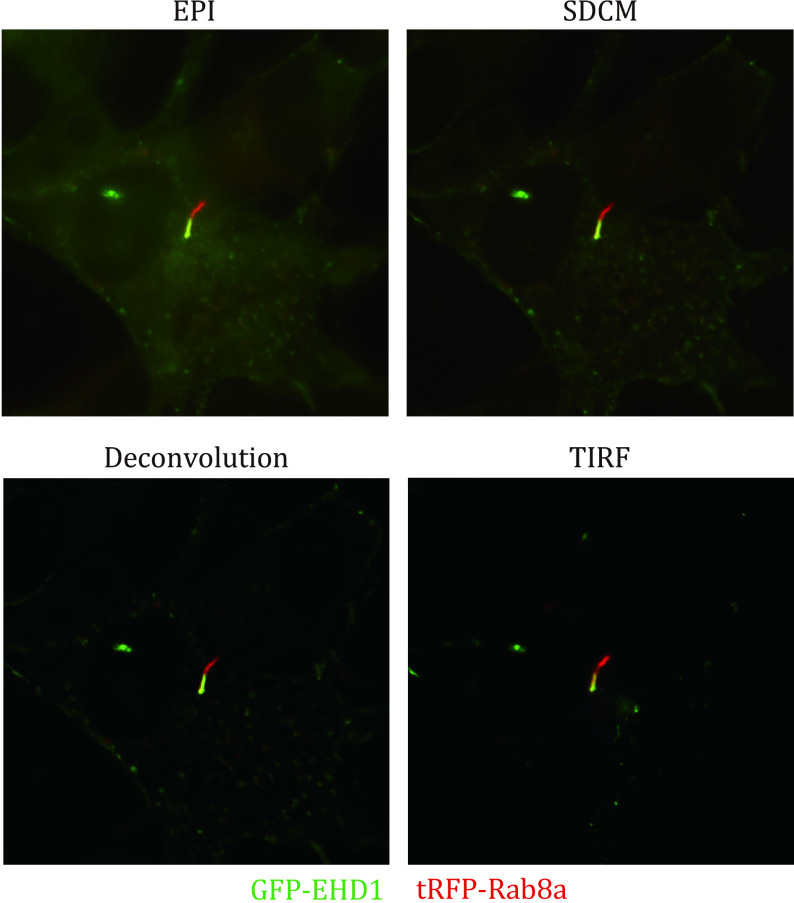
Comparison of epifluorescence, spinning disk confocal, image deconvolution and TIRF microscopies in primary cilia imaging. RPE-1 cells expressing ciliary pocket marker GFP-EHD1 and ciliary membrane marker Rab8a-tRFP were imaged with different microscopy modalities using a Marianas inverted microscope system (Intelligent Imaging Innovations). Note the differences in the signal-to-noise ratio of microscopy techniques. Scale bar, 5 µm

Widefield epifluorescence microscopy is the simplest and cheapest form of live-cell imaging available for imaging ciliary processes. Almost any widefield microscope can be modified to perform live-cell imaging. During epifluorescence imaging, the fluorescent light passes through the sample at a straight angle, and both the illuminated and emitted lights travel through the same objective. Typically, a high-intensity xenon arc or mercury vapor lamp light source is used with narrow wavelength excitation filters to illuminate a sample's fluorescence. Recently, LED light sources have been developed for epifluorescence microscopy, which provides a long-life and high power light source for imaging. Live cell epifluorescence microscopy has been used to investigate ciliogenesis initiating events regulated by preciliary vesicle trafficking (Westlake *et al*. 2011). More recently, this approach was employed to show that the ciliary membrane undergoes a process called cilia decapitation, a process which is thought to eliminate ciliary components to the extracellular space upon growth stimulation (Phua *et al*. 2017). In this study, live-cell imaging was performed using fluorescent protein-tagged 5HT6 and Arl13b to mark the ciliary membrane. Examination of ciliary membrane dynamics during cell division using epifluorescence microscopy with Arl13b-mKate2 or Sstr3-eGFP markers showed that the ciliary membrane attached to the mother centriole was endocytosed at the onset of mitosis and persisted through mitosis at one spindle pole. Notably, the daughter cell that inherited the ciliary membrane retained stem cell character in embryonic neocortical stem cells (Paridaen *et al*. 2013). An issue with using epifluorescence microscopy is that a large sample area is illuminated by excitation light during imaging; the signal from outside the focal plane can produce high background signals in images collected (Fig. 3). Consequently, these images have a lower axial resolution (800 nm) (Stemmer *et al*. 2008), and there can be high photobleaching and phototoxicity associated with this modality. Fluorescence deconvolution software can be employed to improve images by removing out-of-focus signals, but this requires a 3D stack. Consequently, this approach is not well suited for monitoring fast-moving molecules or structures in 3D.

### Laser scanning confocal microscopy (LSCM)

LSCM uses a focused laser beam and a pinhole to block out-of-focus light before signal intensity is collected to visualize fluorescent samples. This feature makes LSCM ideal for imaging thicker samples (Murray 2011). LSCM has been successfully applied to study ciliary IFT trafficking, ciliogenesis and ciliary membrane dynamics in cilia research. For example, the IFT trafficking in *Drosophila* chordotonal neurons was first imaged using a Zeiss LSM 780 LSCM (Lee *et al*. 2018). In another study, *ex vivo* live-cell imaging was used to examine cell division and found that cilia formation and the ciliary sonic hedgehog signaling response are asynchronous between the daughter cells (Piotrowska-Nitsche and Caspary 2012). In a recent study, LSCM live imaging in mIMCD3 cells showed that the predominant mode of cilium loss was via rapid deciliation, in which the membrane and axoneme of the cilium were shed from the cell (Mirvis *et al*. 2019). In another study, LSCM was used to track the kinetics of cell cycle progression and cilia assembly and disassembly in cells and inducible mice (Ford *et al*. 2018). Although LSCM offers several advantages over epifluorescence microscopy on the depth of view, background rejection and axis resolution, this approach's major disadvantages are the relatively slow scanning speed of large view field and the phototoxicity caused by intensive laser light when imaging live cells. Thus, it is more suitable for investigating relatively slow events like cell division and cilia assembly that happen in a timescale of minutes or more.

### Spinning disc confocal microscopy (SDCM)

SDCM is suitable for acquiring images in living cells at high speeds in two or three dimensions. Photobleaching and phototoxicity are typically lower with SDCM than LSCM since a high quantum efficiency CCD or sCMOS camera is used in SDCM instead of a photomultiplier allowing the capture of more images over more extended periods (Fig. 1B and Fig. 3). It has been shown that the movement of primary cilia can promote cilia–cilia encounters in MDCK cells by using SDCM with sampling intervals of a few seconds (Ott and Lippincott-Schwartz 2012). In our work, we carried out live-cell imaging using SDCM to determine the sequence of early ciliogenesis events. In RPE-1 cells expressing ciliary membrane markers EHD1 and Rab8a, we found that EHD1 was associated with the developing ciliary membrane before Rab8a (Lu *et al*. 2015a). We also showed that the ciliary membrane accumulated at the basal body prior to the localization of transition zone proteins. Interestingly, we could also demonstrate that centriole to basal body transition marked by CP110 removal from the mother centriole occurs with distal appendage vesicle docking. Ultimately, the developing intracellular primary cilium has to fuse with the plasma membrane to emerge extracellularly. Traditional ciliogenesis assays can detect cilia length and number, but not the emergence of the cilium from the cell surface. An IN/OUT assay was developed to determine the three stages of primary cilium: inside, outside or partial cilium (Kukic *et al*. 2016). In this assay, pHluorin- (a pH-sensitive GFP variant) tagged Smo was expressed to mark ciliary stages. Since the N-terminal pHluorin tag is only fluorescent when the cilia are exposed to the extracellular environment, it can be used to label emerged cilia in non-permeabilized live cells and imaged with SDCM and other live-cell imaging approaches. SDCM has also been used for live imaging of the cilium in animals. Ford *et al*. developed and characterized a multicistronic biosensor allowing live imaging of cell cycle progression and cilia dynamics in cells and mice (Ford *et al*. 2018). They observed that cilia persist through the G/S transition and into the S/G2/M phase using SDCM in 3T3 cells and mice embryos. Recently, we identified a tubular membrane structure near the basal body following ciliogenesis initiation in cultured RPE-1 cells by SDCM live-cell imaging. We showed that these membrane structures have similar properties *in vivo* in developing zebrafish (Insinna *et al*. 2019a).

### Total internal reflection fluorescence microscopy (TIRFM)

**Figure 4:**
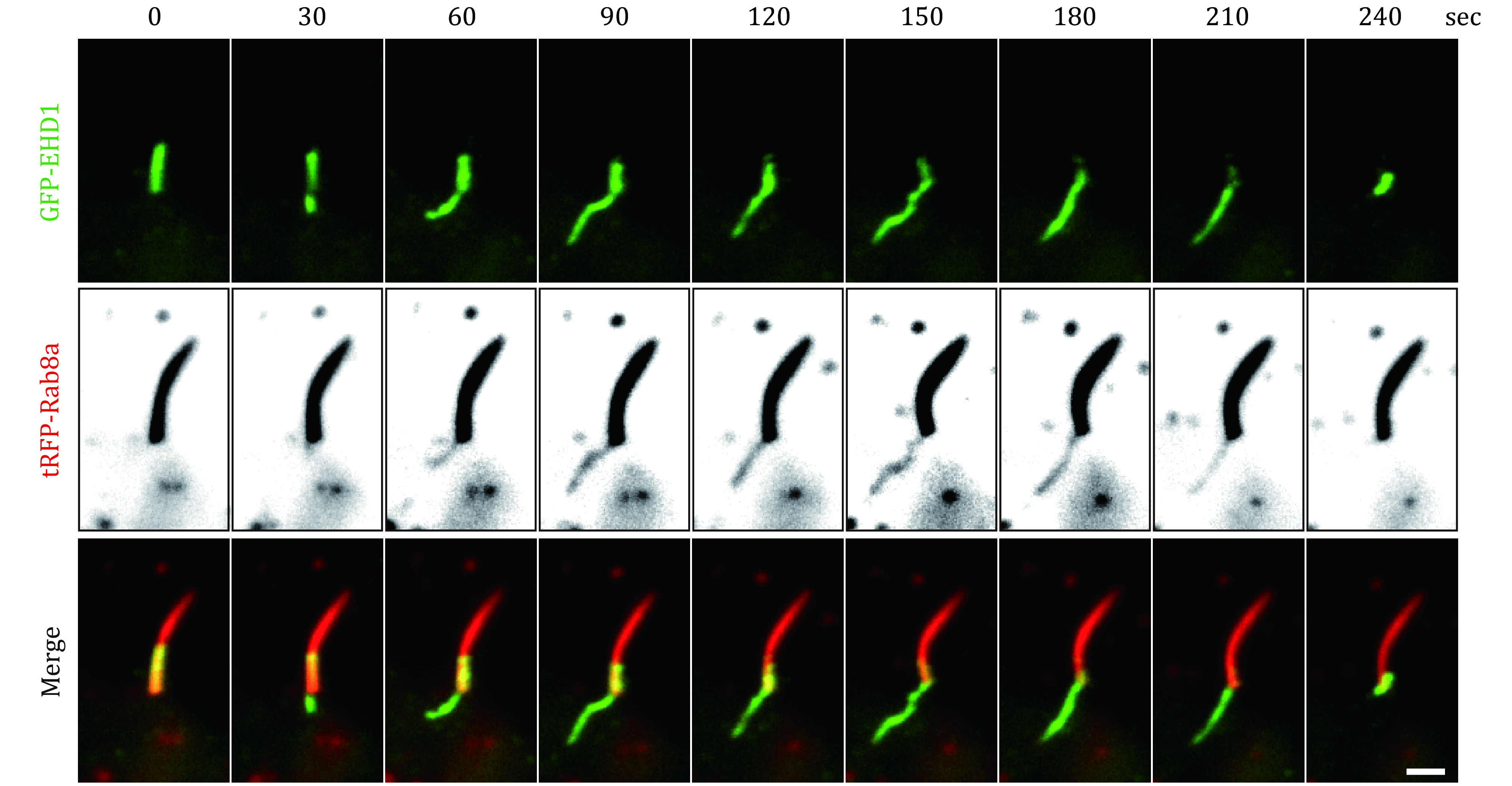
Dynamics of GFP-EHD1 and tRFP-Rab8a at the ciliary pocket and ciliary membrane by TIRFM. Cilium in RPE-1 cells expressing GFP-EHD1 and tRFP-Rab8 was imaged with TIRFM. Note that GFP-EHD1 and tRFP-Rab8a localized on dynamic membrane tubules at the ciliary pocket membrane. The figure showing images captured every 30 sec. The signal intensity of tRFP-Rab8a was inverted and enhanced to demonstrate the presence of Rab8a in the membrane tubules at the base of cilia. Scale bar, 5 µm

TIRFM images a thin region of specimen, usually less than 200 nm above the cover glass can be observed. This imaging technique has a high signal-to-noise ratio as fluorophores 200 nm above the cover glass are not exited. The cilium has a diameter of approximately 250 nm; thus, TIRFM is suitable for live imaging of cilia dynamics when cilia are appressed to cover glass (Fig. 3) (Ishikawa and Marshall 2017; Lechtreck 2016). This approach has been widely applied to study IFT particle movement in flagella and cilia (Ishikawa and Marshall 2015; Jiang *et al*. 2015; Lechtreck 2013). Recently, we imaged the dynamics of GFP-EHD1/RFP-Rab8 positive membrane tubules at the ciliary pocket membrane in RPE-1 cells by TIRFM (Fig. 4) (Insinna *et al*. 2019b).

### Fluorescence recovery after photobleaching (FRAP) Microscopy

FRAP can be used to investigate the dynamics of fluorescent molecule movement in cells. Fluorescent molecules can be bleached selectively in regions of the cell (region of interest or ROI) by exposure to high-intensity light followed by monitoring the recovery of fluorophore-signal to this ROI over time. The fluorophore recovery speed reflects the diffusion speed or turn-over of the molecules in the ROI allowing for lateral diffusion of fluorescently-tagged proteins to be measured. The cilium is particularly suited for this imaging technique as it is highly dynamic and compartmentalized with steady protein trafficking within and to and from this organelle. Although the movement of some ciliary proteins like IFT particles inside a cilium can be directly observed with SDCM or TIRFM, the dynamics of other ciliary proteins like GPCRs are difficult to image using these microscopy techniques due to the background of some of these highly enriched and evenly distributed molecules inside the cilium. Quenching the high background inside the cilium and then watching the reentry of new molecules into the bleached area can reveal information about protein trafficking inside the cilium and the molecular exchange between the cilium and cytoplasm through the connecting transition zone and transition fiber. FRAP studies have revealed high mobility of ciliary membrane proteins within the cilia but a lower exchange rate between cilia and cytoplasm (Chadha *et al*. 2019). In contrast, IFT proteins are rapidly traveling in/out of cilia (Hu *et al*. 2010). Studies in both RPE-1 cells and mouse rod photoreceptors have demonstrated that ciliary opsin movement inside cilium is comparable to that of one of the IFT proteins (IFT88) and is dependent on heterotrimeric kinesin-2 (Trivedi *et al*. 2012). We performed FRAP experiments to compare ciliary trafficking of the hedgehog pathway GPCR Smo in control and Joubert-syndrome and related disorders (JSRD) patients cells caused by CEP290 mutation. This study demonstrated that Smo-GFP transport into the cilium was higher in JSRD patient cilia than in control cells and that the reduced levels of Smo-GFP observed in patient cilia resulted from lower retention in the cilia, possibly caused by higher transport rates out of the cilium (Shimada *et al*. 2017).

## FUTURE PROSPECTIVE

Live-cell imaging techniques have been important for studying the dynamics and function of primary cilia, motile cilia and flagella. Using live-cell imaging to analyze the behavior of cilia requires proper labeling of cilia-specific markers with fluorophores. It is expected that the development of brighter and more stable fluorophores will significantly improve the application of live-cell imaging for studying ciliary processes. In addition, genetically encoded self-labeling tags coupled to structurally-modified synthetic dyes, which have increased quantum efficiency and improved photon yield, will enhance the application of living cell ciliary imaging approaches (Shimada *et al*. 2017). Most of the live-cell imaging analyses of cilia in mammalian cells are currently carried out with exogenously overexpressed markers. The development of CRISPR/Cas9-based homology-directed repair (HDR) or non-homologous end joining (NHEJ) techniques for knocking-in fluorescence reporters to an endogenous genomic locus shed light on future live-imaging of cilia proteins at the endogenous level (He *et al*. 2016). For the most part, the traditional live-cell imaging approaches that have been used to investigate ciliary structure and function have limited spatial resolution. Thus advances in microscopy techniques such as 3D structured illumination microscopy (SIM), Airyscan, and SoRa spinning disk have the potential to provide super-resolution live imaging. Finally, lattice light-sheet microscopy could be used to offer gentler and longer-term live imaging to examine ciliary dynamics. Using these advanced live-cell imaging approaches, together with other microscopy techniques such as electron microscopy will undoubtedly reveal new insight into this critical organelle's functions in normal and diseased states.

## Conflict of interest

Quanlong Lü, Chuanmao Zhang and Christopher J. Westlake declare that they have no conflict of interest.
